# An Innovative Insulin Dose Self-Titration Toolkit for Adults Living With Type 2 Diabetes Mellitus

**DOI:** 10.2196/75903

**Published:** 2025-11-26

**Authors:** Nadin Abbas, Heather Lochnan, Sandhya Goge, Annie Garon-Mailer, Cathy J Sun

**Affiliations:** 1 Faculty of Medicine, University of Ottawa, Ottawa, ON, Canada; 2 Division of Endocrinology and Metabolism, Department of Medicine, The Ottawa Hospital, 1967 Riverside Drive, 4th Floor, Ottawa, ON, K1H 7W9, Canada, 1 6137388333; 3 Ottawa Hospital Research Institute, Ottawa, ON, Canada; 4 Foustanellas Endocrine and Diabetes Centre, The Ottawa Hospital, Ottawa, ON, Canada; 5 School of Nursing, Faculty of Health Sciences, University of Ottawa, Ottawa, ON, Canada

**Keywords:** type 2 diabetes mellitus, outpatient diabetes care, insulin dose self-titration, toolkit, patient education

## Abstract

We developed an innovative bilingual toolkit comprising a personalized action plan and educational videos to encourage insulin dose self-titration by adults living with type 2 diabetes.

## Introduction

“Insulin self-titration” is a strategy where people living with diabetes adjust their insulin doses within parameters recommended by their diabetes care team. While commonly used by people living with type 1 diabetes mellitus, this strategy is remarkably less frequent for people living with insulin-treated type 2 diabetes mellitus (T2D). People living with T2D are less confident with insulin self-titration [1-3]. Studies on insulin self-titration in T2D have been mostly limited to basal insulin [4-9]. Furthermore, although these studies showed that insulin self-titration could be equally or more effective than physician-directed titration [4-10], their interventions required close medical surveillance which could be challenging for long-term and widespread implementation in diabetes clinics [9]. The objective of this feasibility study was to develop an educational toolkit on insulin dose self-titration for people living with T2D.

## Methods

### Ethical Considerations

The Ottawa Health Science Network Research Ethics Board exempted this study (on 18 November 2024) as quality improvement. Furthermore, the contents of the toolkit abide by current clinical care guidelines. Participants provided informed voluntary consent. Participants’ survey responses were collected anonymously, and no compensation was provided.

### Study Design

**Figure 1.**
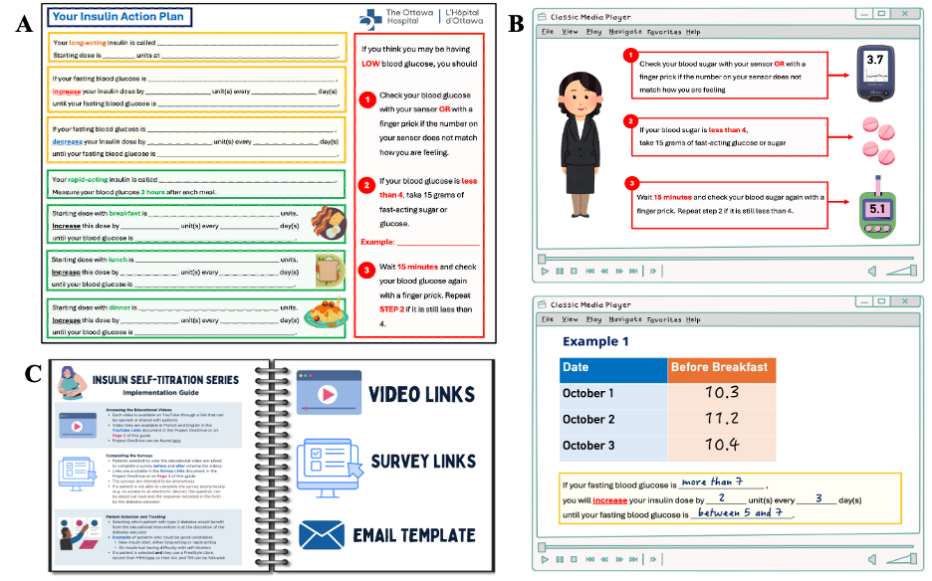
The customizable Insulin Action Plan showing (A) titration of long-acting insulin and rapid-acting insulin and hypoglycemia management. Samples from videos: hypoglycemia management (B, top), long-acting insulin titration (B, bottom). Implementation guide for healthcare providers (C).

This was a quality improvement initiative at the diabetes clinics of a Canadian academic tertiary care center. We developed a toolkit in English and French (Figure 1). The toolkit included a customizable insulin dose self-titration guide, “Insulin Action Plan” (Multimedia Appendix 1), which included information on hypoglycemia recognition and management. Certified diabetes educators (CDEs), who are part of the circle of care, tailored this guide using their medical directives for insulin dose adjustment recommendations within 20% of the current dose for each person living with T2D. The CDEs also provided web site links (Multimedia Appendix 1) to educational videos containing examples on how to apply the Insulin Action Plan. Toolkit development was informed by diabetes care guidelines with feedback incorporated from CDEs and people living with T2D. Exclusion criteria were known cognitive impairment or known history of hypoglycemia unawareness. Online pre- and post-toolkit anonymous surveys were sent (via Google Forms) to participants 1 week apart to rate their self-assessed understanding of insulin self-titration on a scale of 1 to 10.

## Results

Table 1 summarizes data obtained from participants who completed the pre- (n=15) and post-toolkit (n=10) self-assessments of their comprehension of insulin dose self-titration. The median score increased from 3 (IQR 1‐7) in the pre-toolkit responses to 8 (IQR 6‐9) in the post-toolkit responses. A Mann-Whitney *U* test indicated a statistically significant increase in the subjective understanding of insulin dose self-titration (U=27, Z=-2.63, *P*=.004), with a large effect size (*r*=0.53).

**Table 1.** Participants’ self-assessment survey results.

Survey question	Response to survey question	
On a scale of 1 to 10 (where 1 is no understanding and 10 is complete understanding), how would you rate your understanding of how to titrate your insulin dose?	Before insulin dose self-titration toolkit (n=15)	After insulin dose self-titration toolkit (n=10)
No understanding (1)	5	-
Little understanding (2-3)	4	1
Some understanding (4-6)	2	3
Good understanding (7-9)	3	6
Complete understanding (10)	1	-

## Discussion

In this feasibility study, people living with insulin-treated T2D were generally accepting of this toolkit, which was easy to integrate into routine clinical care. The online delivery of this toolkit is accessible to participants all day, every day. Therefore, it is worthwhile to proceed to a full study that will aim to address this feasibility study’s many limitations.

The main strength of this innovative toolkit is that, once optimized, minimal ongoing resources will be required. The “Insulin Action Plan” is easy to personalize and update for each participant. This toolkit was co-created with CDEs and people living with T2D to be easily integrated into routine care. The toolkit’s freely accessible online educational videos with concrete examples of insulin dose self-titrations can be viewed as often as needed to refresh and solidify understanding of this concept.

The major limitations of this feasibility study include the lack of sociodemographic data collection, objective assessment regarding whether these people living with T2D correctly self-titrated their insulin doses or the eventual impact on glycated hemoglobin, time-in-range, and hypoglycemia occurrence. The generalizability of this feasibility study’s results is limited by the small sample size and short follow-up.

The next phase of this innovative toolkit will include the collection of sociodemographic data and longitudinal follow-up to determine outcomes including the accuracy of participants’ insulin dose self-titrations, as well as their impact on their glycated hemoglobin, time-in-range, and hypoglycemia occurrences. We would anticipate an improvement in these determinants of glucose management with successful insulin dose self-titration [4-7]. Future directions for this initiative would be a full study recruiting participants reflecting the diversity of people living with T2D, ongoing gathering of feedback from people living with T2D, and evaluating objective glycemic measures with longitudinal follow-up.

## Supplementary material

10.2196/75903 Multimedia Appendix 1 Web site links for the toolkit components.

## Abbreviations

CDE: certified diabetes educator
T2D: type 2 diabetes mellitus
