# Supplementary material for: An Innovative Insulin Dose Self-Titration Toolkit for Adults Living With Type 2 Diabetes Mellitus
Source: JMIR Diabetes. 2025 Nov 26;10:e75903. doi: 10.2196/75903 (PMC12661589; doi:10.2196/75903)
Supplement: Multimedia Appendix 1 [file diabetes-v10-e75903-s001.pdf]

Research Letter re-submission to *JMIR Diabetes*

Title: An innovative insulin dose self-titration toolkit for adults living with type 2 diabetes mellitus

Supplemental Materials File: Internet web site links that we will maintain and keep active.

Insulin Action Plan (self-titration guide) English and French versions:

https://drive.google.com/drive/folders/1jbAHXeCYhzDueZGOzhwydDGqR_TTLyOk

Web site links for the videos

Introduction to insulin:

English: https://youtu.be/QjG5a9uuC-I

French: https://youtu.be/kJkiXYuoqE8

Long-acting insulin dose titration:

English: https://youtu.be/OipNxQpAnXo

French: https://youtu.be/wkZO30nnOk0

Rapid-acting insulin dose titration

English: https://youtu.be/8CK8y-J0xh4

French: https://youtu.be/-Tb7-lhE1Fo

Pre-mixed insulin dose titration:

English: https://youtu.be/yVKITDreSc0

French: https://youtu.be/cHWvjakJSdI

Hypoglycemia

English: https://youtu.be/yFUpa-_X3_A

French: https://youtu.be/HUnY-W4TN9Y
